# Rupture of the external iliac and bilateral femoral arteries in a patient with anorexia nervosa

**DOI:** 10.1007/s40519-025-01789-2

**Published:** 2025-09-26

**Authors:** Kan Goto, Takeaki Sato, So Sampei, Yasuhiro Sato, Motoyori Kanazawa, Shigeki Kushimoto, Shin Fukudo

**Affiliations:** 1 Department of Psychosomatic Medicine, Tohoku University Hospital, 1-1 Seiryo-Machi, Aoba-Ku, Sendai, 980-8574 Japan; 2 Department of Emergency and Critical Care Medicine, Tohoku University Hospital, Sendai, Japan; 3 Department of Behavioral Medicine, Tohoku University Graduate School of Medicine, Sendai, Japan; 4 Kawasaki Saiwai Hospital, Kawasaki, Japan

**Keywords:** Anorexia nervosa, Vascular complications, Vascular fragility, Arterial rupture, Malnutrition

## Abstract

**Background:**

Severe anorexia nervosa can result in life-threatening systemic complications. However, arterial rupture in the context of anorexia nervosa has not been reported.

**Case report:**

A 31-year-old woman who had developed anorexia nervosa in her early teens and had extreme malnutrition with a body mass index of 8.5 kg/m^2^ was admitted to our hospital with impaired consciousness due to hypoglycemia. During the management of hypoglycemia and refeeding syndrome, the patient developed hematochezia and shock. Contrast-enhanced computed tomography revealed rupture of the right external iliac and bilateral femoral arteries. Nonocclusive mesenteric ischemia was also detected. Surgical interventions for the ruptured arteries and the intestinal lesion were not indicated due to her poor condition with coagulopathy, as well as the presumed vascular fragility suggested by the presence of multiple arterial ruptures. The patient died later that day.

**Conclusions:**

This case highlights a previously unreported and fatal vascular complication of anorexia nervosa. Extreme malnutrition may cause vascular fragility, increasing the risk of arterial rupture due to structural collapse of the vessel wall. Clinicians should be aware of this rare but life-threatening complication when treating patients with severe anorexia nervosa.

**Level of evidence:**

Level V, case report.

## Introduction

Patients with anorexia nervosa often present with systemic complications, including cardiovascular, cutaneous, gastrointestinal, endocrine–metabolic, hematological, neurological, and respiratory disorders [1]. A recent meta-analysis found that the weighted mortality rate (i.e., deaths per 1000 person-years) was 5.1, which is higher than that of other psychiatric or psychosomatic disorders [2]. Cardiovascular complications are estimated to account for at least 30% of deaths [3]. Although various cardiovascular complications have been reported, including bradycardia, hypotension, arrhythmias, repolarization abnormalities, congestive heart failure, myocardial infarction, and sudden death [3], arterial rupture has not been documented.

Here, we report a patient with anorexia nervosa who experienced arterial rupture after prolonged and severe malnutrition. We also discuss the possible vascular structural changes induced by chronic undernutrition and emphasize the need for clinical vigilance in managing patients with anorexia nervosa, given the potential for unexpected and fatal vascular events.

## Case report

The patient was a 31-year-old woman (height 1.54 m, weight 20.2 kg, and body mass index 8.52 kg/m^2^) who had developed anorexia nervosa in her early teens. Her body mass index remained below 15 kg/m^2^ for over 10 years, dropping below 10 kg/m^2^ during the past year. She first visited our department at the age of 30 years after treatment in departments of psychiatry and internal medicine at other hospitals. She had a strong desire to lose weight and a fear of gaining weight. She had repeatedly been in and out of the hospital in a short period, and was unable to adapt to treatment. Nasogastric tube feeding was initiated due to inadequate oral intake; however, the patient was non-compliant with the nutritional therapy. Her daily caloric intake remained below 300 kcal. Laboratory tests revealed a progressive deterioration in liver function, prolonged hypoglycemia, and hyponatremia, all of which were attributed to severe malnutrition.

She was brought to our hospital by ambulance due to impaired consciousness. On arrival, her vital signs were as follows: temperature 36.2 °C, heart rate 61 beats/min, respiratory rate 18 breaths/min, blood pressure 85/56 mmHg, and oxygen saturation 100% (on 6 L/min oxygen via face mask). Her level of consciousness was assessed as a Glasgow Coma Scale (GCS) score of 6 (eye opening 1, best verbal response 1, best motor response 4). Physical examination revealed bilateral miosis, delayed pupillary light reflexes, and cold extremities. Her blood pressure had been consistently in the 80/50 mmHg range during prior hospitalizations and outpatient visits; thus, she was not considered to be in shock.

An arterial blood sample was obtained from the right inguinal region to assess her condition. Initial blood tests revealed elevated liver enzymes, thrombocytopenia, hyponatremia, hypocalcemia, hypochloremia, hyperphosphatemia, hypolipidemia, hypocholinesterasemia, and hypoproteinemia (Table 1). Blood glucose levels were undetectable. Arterial blood gas analysis showed no evidence of hyperlactatemia. Whole-body computed tomography (CT) demonstrated severely reduced skeletal muscle mass and adipose tissue, without any intracranial or other focal lesions to explain the altered consciousness. The electrocardiogram revealed a normal sinus rhythm; however, low potentials were noted in all leads. Based on these findings, she was diagnosed with hypoglycemic coma. Vitamin B1 and 40 mL of 50% glucose solution were administered. Her blood glucose level increased to 568 mg/dL, and her level of consciousness improved to a GCS score of E4V4M6.

**Table 1:** Laboratory data upon admission

[Hematology]			[Biochemistry]		
White blood cell	6.6 × 10^3^	/μL	Total protein	5.1	g/dL
Neutrophil	5.2 × 10^3^	/μL	Albumin	3.3	g/dL
Lymphocyte	1.2 × 10^3^	/μL	Urea nitrogen	32.0	mg/dL
Red blood cell	3.87 × 10^6^	/μL	Creatinine	0.55	mg/dL
Hemoglobin	12.4	g/dL	Total bilirubin	3.7	mg/dL
Platelet	114 × 10^9^	/L	Aspartate aminotransferase	1,290	U/L
			Alanine aminotransferase	621	U/L
[Arterial blood gas]			Alkaline phosphatase	98	U/L
pH	7.333		Cholinesterase	109	U/L
pCO_2_	44.6	mmHg	Triglycerides	7	mg/dL
pO_2_	275	mmHg	Total cholesterol	146	mg/dL
HCO_3_	23	mEq/L	Sodium	123	mEq/L
Lactate	1.1	mmol/L	Potassium	3.7	mEq/L
FiO_2_	0.5		Chloride	94	mEq/L
			Phosphorus	4.9	mg/dL
			Magnesium	2.4	mg/dL
			Calcium	7.5	mg/dL
			C-reactive protein	0.01	mg/dL

She was admitted to the emergency and critical care center for comprehensive management. An arterial line was placed in the right radial artery to closely assess hemodynamics due to the high risk of sudden change. To prevent refeeding syndrome, enteral and parenteral nutrition with approximately 5 kcal/kg/day was initiated. However, blood tests on the same day revealed a decrease in phosphate levels from 4.9 to 2.9 mg/dL, prompting the initiation of intravenous phosphate supplementation. Frequent episodes of hypoglycemia were observed, and 50% glucose was administered repeatedly.

On the day after admission, her blood pressure was stable in the 80/50 mmHg range and the arterial line was removed on hospital day 2. However, not only the prolonged hypophosphatemia. but also hypomagnesemia and other abnormalities required cautious management due to the risk of refeeding syndrome. Therefore, she was closely monitored with daily blood tests. Peripheral venous blood sampling was attempted. but was difficult due to the narrow vessel lumen. The femoral vein was also difficult to access for the same reason, so blood samples were taken from the bilateral femoral arteries using a 22G needle. After each blood draw, adequate manual compression was applied to achieve hemostasis.

**Fig. 1:**
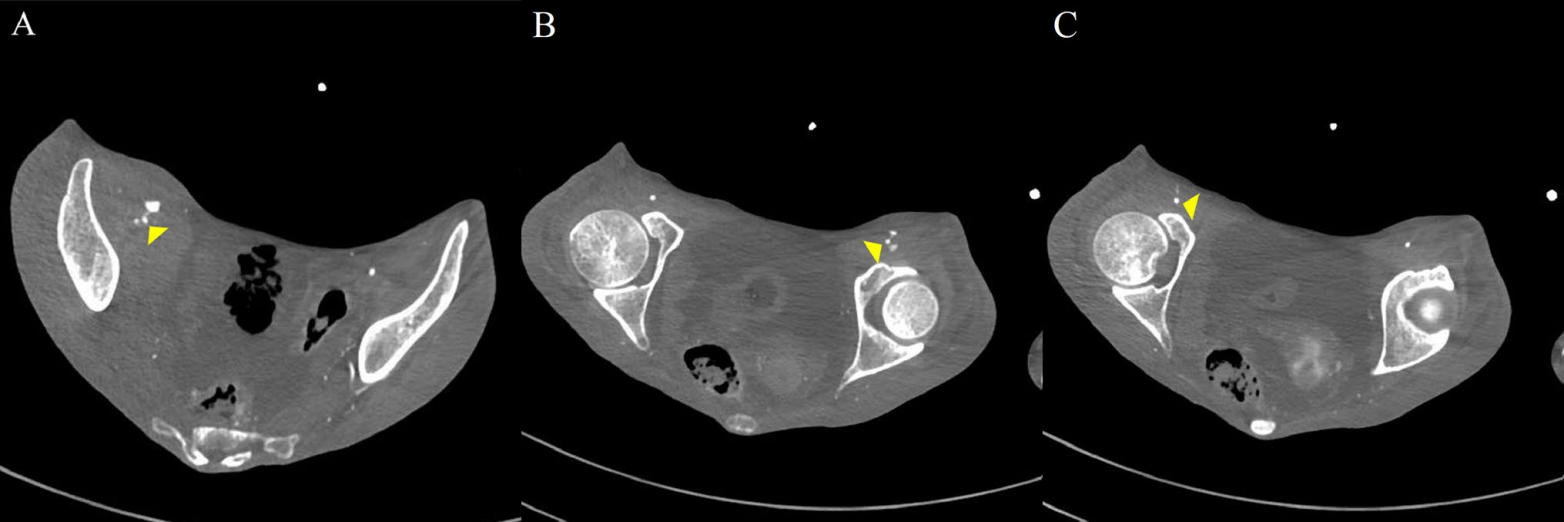
Pelvic contrast-enhanced computed tomography. **A** Contrast extravasation from the right external iliac artery during the arterial phase (yellow arrow). **B** Contrast extravasation from the left femoral artery during the arterial phase (yellow arrow). **C** Contrast extravasation from the right femoral artery during the arterial phase (yellow arrow)

On hospital day 7, dark red stools were noted. Her systolic blood pressure dropped to 50 mmHg, and her heart rate increased to 100 beats/min. Due to the presence of persistent bloody stools, hemorrhagic shock secondary to gastrointestinal hemorrhage was suspected. Contrast-enhanced abdominal CT was performed to identify the bleeding source and revealed extravasations of contrast medium from the right external iliac artery and bilateral femoral arteries in the arterial phase (Fig. 1). Based on these findings, ruptures of the right external iliac artery and bilateral femoral arteries were diagnosed. The CT also showed reduced contrast enhancement in the bowel wall from the cecum to the ascending colon and gas in the hepatic portal vein, without evidence of vascular occlusion, findings that were also strongly suggestive of nonocclusive mesenteric ischemia. Blood tests revealed thrombocytopenia and coagulopathy (platelets 8 × 10^9^/L, prothrombin time international normalized ratio > 10, activated partial thromboplastin time > 180 s, and fibrinogen 37 mg/dL). Surgical interventions for the vascular and intestinal lesions were not indicated due to her poor general condition, coagulopathy, and multiple ruptures of medium-sized vessels. She was managed with blood transfusion and hemostasis through compression; however, she died later that day.

## Discussion

Arterial rupture has not been reported in association with anorexia nervosa. Known causes of arterial rupture include true aneurysms, pseudoaneurysms, and spontaneous rupture associated with connective tissue disorders, or congenital arterial anomalies [4]. In our case, CT imaging revealed no evidence of aneurysmal or pseudoaneurysmal changes, and the patient had neither a history nor clinical features suggestive of heritable vascular disorders. We cannot rule out the possibility that repeated blood sampling from the femoral artery influenced the vascular condition. However, rupture occurred in multiple medium-sized arteries, including the right external iliac artery, which had never been punctured. Furthermore, despite repeated access at the right radial artery for arterial line placement, no bleeding or structural vascular abnormalities, such as pseudoaneurysm or dissection, were observed at that site. These findings indicate that arterial puncture alone cannot fully explain the pathology. Vascular fragility potentially related to prolonged and severe malnutrition should therefore be considered as a contributing factor.

Severe anorexia nervosa is often associated with arterial atherosclerotic changes [5]. Early vascular stiffening—evidenced by elevated cardio-ankle vascular index and increased total peripheral resistance, even in adolescent patients—has also been documented [6]. The condition is further characterized by endocrine dysfunction, micronutrient deficiencies, and elevated inflammatory cytokines, which may contribute to vascular smooth muscle and endothelial dysfunction [7, 8].

Endocrine dysfunction in anorexia nervosa, such as decreased levels of triiodothyronine and estradiol, is thought to impair vascular health, as triiodothyronine contributes to the maintenance of endothelial and vascular smooth muscle function and estradiol plays a protective role in the vascular endothelium [7].

In addition, patients with anorexia nervosa frequently present with deficiencies in micronutrients such as zinc, selenium, and vitamin C. These micronutrients are involved in the regulation of endothelial inflammation (zinc and selenium) and in the development and structural integrity of endothelial cells and the vascular basement membrane (vitamin C) [7].

Inflammatory activity may also contribute to vascular pathology in these patients. One study reported that individuals with anorexia nervosa tend to have elevated circulating levels of interleukin-6 and tumor necrosis factor-α, along with enhanced leukocyte–endothelial interactions, including reduced rolling velocity, increased rolling flux, and increased firm adhesion, indicating a pro-inflammatory and pro-atherogenic endothelial phenotype [8].

Taken together, these pathophysiological factors may collectively result in endothelial dysfunction and weakening of the intimal layer, thereby increasing the risk of vascular fragility in patients with anorexia nervosa. While histopathological examination was not available in this case, the clinical presentation is consistent with vascular fragility due to these malnutrition-related changes. A report of digital necrosis following arterial puncture for blood gas analysis in a patient with anorexia nervosa also supports the presence of vasculopathy [9]. Additionally, a case of right thyrocervical trunk rupture after right internal jugular vein puncture highlights the likelihood of vascular abnormalities in patients with anorexia nervosa, as the connective tissue of the vascular wall may be considerably weakened due to starvation and energy deprivation, making vascular structures vulnerable [10]. Overall, these findings suggest that chronic and severe malnutrition, extreme underweight, and prolonged illness played important roles in the development of vascular fragility in this case. We speculate that fragility of the arterial wall due to severe anorexia nervosa may have contributed mainly to structural collapse of the arterial wall, ultimately resulting in rupture.

This case not only underscores the critical need for heightened awareness of vascular fragility in patients with severe anorexia nervosa but also suggests that patients with extreme malnutrition may develop life-threatening complications that are not typically anticipated in the management of other conditions. Clinicians should remain vigilant for potential vascular complications in patients with severe anorexia nervosa and exercise caution in treatment, considering the possibility of various complications.

## Strengths and limitations

This is the first report to describe medium-sized arterial rupture in a patient with anorexia nervosa. Histopathological examination was not performed, limiting the ability to assess the underlying vascular pathology.

### What is already known on this subject?

Anorexia nervosa is associated with a wide range of systemic physical complications, some of which can be life threatening.

### What this study adds?

This is the first report to suggest a possible link between severe and prolonged malnutrition in anorexia nervosa and vascular complications. Clinicians should recognize that vascular complications may occur in patients with anorexia nervosa and remain vigilant during clinical management.

## Data Availability

No datasets were generated or analyzed during the current study.
